# Targeted Use of Antimicrobials in Periodontal Therapy

**DOI:** 10.7759/cureus.79874

**Published:** 2025-03-01

**Authors:** Sarah Zubair

**Affiliations:** 1 General Dentistry, Royal College of Surgeons of England, London, GBR; 2 Restorative and Aesthetic Dentistry, Freshdental Institute, London, GBR; 3 Dentistry, CMH Lahore Medical College and Institute of Dentistry, Lahore, PAK; 4 Restorative and Aesthetic Dentistry, Royal College of Surgeons of England, London, GBR

**Keywords:** antimicrobial therapy, generalized periodontitis, localized aggressive periodontitis, nonsurgical periodontal therapy, periodontal pathogens, s: chronic periodontitis

## Abstract

Periodontitis is a widespread health issue that requires effective prevention and management due to its increasing prevalence and impact on systemic health. The chronic inflammation associated with periodontitis also worsens conditions like diabetes and cardiovascular diseases. Treatment approaches have evolved in response, incorporating local and systemic antimicrobials as adjuncts to traditional mechanical debridement. This literature review explores the role of antimicrobials in periodontal therapy, examining the efficacy of systemic and local options as adjuncts to mechanical debridement. Findings suggest that systemic antimicrobials, especially amoxicillin-metronidazole combinations, benefit aggressive periodontitis cases; their use should be limited to specific, severe cases to mitigate antimicrobial resistance risks. Local antimicrobials provide a more targeted treatment option with reduced systemic side effects, which is particularly useful in managing localized infections. Adjunctive local antimicrobials with scaling and root planning (SRP) significantly reduced probing pocket depths (PPDs) and improved clinical attachment levels (CALs) in periodontal treatment with minimal adverse effects. Tetracycline fibers, minocycline, and doxycycline were most effective in PPD reduction, while chlorhexidine showed the greatest CAL gains. Further research is essential to establish clear guidelines and long-term outcomes in antimicrobial use for periodontal disease management.

## Introduction and background

Periodontitis is defined as a chronic, multifactorial inflammatory gum disease primarily caused by the accumulation of dental plaque, leading to the irreversible destruction of the supporting structures of the teeth; it is a prevalent global disease affecting approximately 10% of the population [1], with severe cases often linked to various systemic conditions such as diabetes, cardiovascular diseases, stroke, rheumatoid, and pulmonary disease [2]. Characterized by a polymicrobial infection, periodontitis can lead to significant periodontal tissue destruction, tooth loss, and reduced quality of life [3]. 

Approximately 600 bacterial species contribute to the balance of host-microbial interactions in the oral cavity, which is critical in maintaining health or promoting disease [4]. Periodontal diseases primarily include gingivitis, a reversible plaque-induced inflammation, and periodontitis, an irreversible infection that leads to periodontal attachment loss [5].

In 2017, the American Academy of Periodontology (AAP) and the European Federation of Periodontology (EFP) introduced an updated classification system, incorporating a staging and grading framework. Staging reflects the severity of periodontal tissue destruction, while grading estimates the rate of disease progression, with stages ranging from I (mild) to IV (severe) and grades from A (slow progression) to C (rapid progression). This classification guides treatment strategies, including using antimicrobials based on disease severity and progression risk [6]. The studies included in this article have used the terms chronic periodontitis (CP) and aggressive periodontitis (AgP) when discussing systemic antibiotics in relation to periodontitis, based on the 1999 classification system [6]. However, the 2017 World Workshop classification has replaced these terms with a staging and grading framework for periodontitis [6]. The studies refer to moderate or deep periodontal sites, which can be aligned with the updated classification. In the 2017 World Workshop on the Classification of Periodontal and Peri-implant Diseases and Conditions, aggressive periodontitis was reclassified and incorporated into a more comprehensive framework. The classification now recognizes periodontitis as a single disease entity with varying degrees of severity and progression. It is now part of the broader category of periodontitis, which is classified into the following groups: Periodontitis Stage I (Initial Periodontitis), Periodontitis Stage II (Moderate Periodontitis), Periodontitis Stage III (Severe Periodontitis with Potential for Tooth Loss), and Periodontitis Stage IV (Advanced Periodontitis with Significant Tooth Loss) accumulation [6]. Aggressive periodontitis, which is a severe form of gum disease characterized by rapid destruction of the tissues supporting the teeth in a relatively younger patient without significant plaque accumulation, is now categorized as stage III or IV generalized periodontitis with grade C, indicating rapid progression [7].

It was observed that despite surgical and mechanical debridement with very strict oral hygiene measures, certain periodontitis patients still experienced periodontal tissue destruction [8]. This led to the development of the concept of adjunctive use of systemic antimicrobials in addition to the standard periodontal therapy regimes in the late 1970s [8]. Initially, antibiotics were used postsurgery to prevent infection [8]. Still, short courses of high-dose systemic antimicrobials have been employed in recent decades alongside mechanical debridement to target specific bacterial pathogens responsible for recurrent disease. This shift toward targeted antimicrobial therapy aims to reduce specific bacteria, potentially eliminating the need for more invasive surgical interventions [8].

Both systemic and local antimicrobials have since been recognized as valuable adjuncts to mechanical debridement [9]. However, recent guidelines emphasize antimicrobial stewardship, recommending antibiotics selectively, particularly in severe or unresponsive cases [10]. This review synthesizes the latest evidence on systemic and local antimicrobials, focusing on the amoxicillin-metronidazole combination for aggressive periodontitis and the benefits of localized, high-concentration therapy for persistent infections. 

## Review

Methods 

This literature review assesses the efficacy and safety of systemic and local antimicrobials in treating periodontal disease. A comprehensive search of electronic databases, including PubMed, Cochrane Library, and the National Elf Service, was conducted with key terms such as "periodontal disease," "chronic periodontitis," "aggressive periodontitis," "antimicrobials," "local drug delivery," and "systemic antimicrobials.” A total of 26 studies were included with in-depth screening, out of which three were systematic reviews and three were systematic reviews and meta-analyses. 

Time Frame

Studies published between January 1999 and December 2023 were considered.

Language

Only articles published in English were included.

Inclusion Criteria

Randomized controlled trials (RCTs), systematic reviews, meta-analyses, literature reviews, observational studies, and recent guidelines focused on adult patients with periodontal disease were included.

Exclusion Criteria

In vitro studies, case reports, conference abstracts, and studies on nonperiodontal conditions were excluded.

Initial screening was performed on titles and abstracts, followed by a full-text eligibility assessment. Relevant data on study design, sample size, antimicrobial type, treatment protocols, and outcome measures were extracted.

Results

The literature review demonstrated that systemic antimicrobials, particularly the amoxicillin metronidazole combination, significantly improve chronic and aggressive periodontitis. For CP, a meta-analysis by Sgolastra et al. showed clinical attachment level (CAL) gain (weighted mean difference (WMD) = 0.21 mm) and probing pocket depth (PPD) reduction (WMD = 0.43 mm) [11]. In aggressive periodontitis, Mendes et al. reported PPD reductions with amoxicillin-metronidazole adjunctive therapy, though CAL gains were minimal [12].

Local antimicrobials as adjuncts to scaling and root planing (SRP) yielded modest improvements. Matesanz-Pérez et al.’s analysis showed significant PPD reduction (WMD = 0.407 mm) and CAL gain (WMD = 0.310 mm) [13]. Tetracycline fibers had the highest PPD reduction (0.727 mm), while chlorhexidine with xanthan gel showed the greatest CAL gain (0.9 mm). Herrera et al. found similar results with PPD reduction (WMD = 0.365 mm) and CAL gain (WMD = 0.263 mm) [14]. Adverse effects with local antimicrobials were mild, primarily involving taste disturbances and gingival erythema [14]. Adverse effects of systemic antimicrobials can include severe reactions such as anaphylaxis, pseudomembranous colitis, or allergic responses to penicillin [15]. This table summarizes the findings from key studies evaluating systemic and local antimicrobial therapies for periodontal disease (Table 1).

**Table 1:** Summary of key findings on antimicrobial efficacy in the treatment of chronic and aggressive periodontitis AL: attachment level (a parameter used to assess periodontal conditions); SRP: scaling and root planing (a common treatment for gum issues); CAL: clinical attachment level (measures periodontal tissue loss in periodontitis. It is calculated by adding pocket depth to gingival recession); PPD: pocket probing depth (the distance from the gum margin to the bottom of the gingival pocket); WMD: weighted mean difference (a method to compare treatment effectiveness by averaging results from multiple studies, emphasizing those with larger sample sizes and more reliable data for evidence-based decision-making)

Study	Condition	Study type	Antimicrobial type	Outcome measures	Key findings
Sgolastra et al. [11]	Chronic periodontitis	Systematic review and meta-analysis	Systemic (amoxicillin-metronidazole combination)	CAL gain (WMD = 0.21mm); PPD reduction (WMD = 0.43mm)	Supports adjunctive use in chronic cases; minimal effect on BOP and suppuration
Mendes et al. [12]	Aggressive periodontitis	Systematic review and meta-analysis	Systemic (amoxicillin-metronidazole combination)	Significant PPD reduction; minimal CAL gain	Effective for PPD reduction in aggressive cases; limited CAL gain
Matesanz-Perez et al. [13]	Chronic periodontitis	Systematic review	Local various *(tetracycline fibers, chlorhexidine with xanthan gel; chlorhexidine with locally administered metronidazole; sustained-release minocycline and doxycycline)	PPD reduction (WMD = 0.407mm); CAL gain (WMD = 0.310mm)	Significant improvements in deep or recurrent pockets; tetracycline fibers showed the highest PPD reduction
Herrera et al. [16]	Periodontitis (general)	Systematic review	Local (various)*	PPD reduction (WMD = 0.365mm); CAL gain (WMD = 0.263mm) at 6 and 9 months	Consistent improvements in PPD and CAL with local antimicrobials; minimal adverse effects
Teughels et al. [17]	Chronic and aggressive periodontitis	Systematic review and meta-analysis	Systemic (amoxicillin-metronidazole combination)	PPD reduction (WMD = 0.448mm); CAL gain (WMD = 0.389mm) at 6 months	Significant improvement in PPD and CAL in deep pockets, with sustained results over one year
Haffajee et al. [18]	Chronic and aggressive periodontitis	Systematic review	Systemic antibiotics	Improved AL (p < 0.001) with SRP; borderline benefit with SRP + surgery or monotherapy	Improved attachment levels in both chronic and aggressive cases; greater effect in aggressive periodontitis

Discussion

This literature review was conducted to evaluate whether antimicrobials play an important role in treating periodontal disease. Compared to other studies, our review highlighted that while systemic and local antimicrobials are effective as adjuncts to SRP, their use should be tailored to specific cases to maximize benefits and minimize risks. A total of 26 studies were included in this review, comprising three systematic reviews and three systematic reviews with meta-analyses, ensuring that our findings are based on robust evidence and aligned with the latest guidelines. This comprehensive evaluation provides valuable insights into optimizing periodontal therapy with adjunctive antimicrobials.

Periodontitis is a complex polymicrobial disease that has been linked to certain bacteria that cause recurrent disease [9]. Moreover, tetracycline-resistant strains like *Actinobacillus actinomycetemcomitans *and *Porphyromonas gingivalis *have been associated with aggressive periodontitis [9]. With time, the pathogens residing in the periodontal pockets seep deeper into the periodontal tissues and, hence, become hard to clean unless access is improved by periodontal surgery [9]. Hence, the traditionally employed most common method of nonsurgical treatment of periodontal disease, i.e., mechanical debridement by SRP only, may fail to remove these complex organisms residing in deep pockets, furcation areas, and subgingival plaque biofilms. Systemic antimicrobials can enter the deeper periodontal tissues and pockets and suppress the pathological activity of the plaque-residing microbes, leading to a reduced bacterial load, which can delay further recolonization and progression of the disease process [9,15].

In the last 30 years, dentists have recently embraced the concept of reducing specific bacterial organisms that cause recurrent disease activity by using a short course of high-dose systemic antimicrobials and mechanical debridement [8]. The main feature of the new approach is the plaque-specific hypothesis, which focuses on reducing the number of specific bacteria that cause recurrent periodontal breakdown [8]. This new treatment paradigm can prevent patients from undergoing more expensive, labor-intensive surgical procedures that were traditionally employed for periodontal therapy, thereby eliminating the need for complex surgery for therapy [8].

Recently, local antimicrobials have been recognized as promoting focused healing in disease-specific sites by localized high-drug concentration administration without causing the side effects associated with systemic antimicrobials, such as antimicrobial resistance [19].

While much progress has been made in understanding the pathogenesis of periodontitis, clear guidelines on antimicrobial selection remain elusive. Routine pathogen susceptibility testing is not commonly performed, adding variability to treatment decisions. Recent guidelines emphasize antimicrobial stewardship, recommending antibiotics only when the benefits outweigh the risks and reserving their use for severe or unresponsive cases [9,10].

Systemic Antimicrobials in Periodontal Therapy

Systemic antimicrobials significantly improve clinical outcomes when used with subgingival scaling, particularly in deep or moderately deep pockets in periodontitis [16,20]. A systematic review by Teughels et al., based on 28 RCTs, showed significant reductions in PPD and gains in CAL after six months and one year [17]. Of these RCTs, 24 were included in the meta-analysis, which concluded that the use of systemic antimicrobials resulted in a significant decrease of full-mouth PPDs and showed reduced PPD value of (WMD = 0.448) and resulted in gain in clinical attachments levels resulting in CAL gain value of (WMD = 0.389mm) when evaluated six months after treatment as compared to control group who were treated with SRP alone [17]. These values showed stability over the one-year period with PPD reduction value (WMD = 0.485 mm) and CAL value (WMD = 0.285 mm); however, some of studies that were included in the meta-analysis had a six-month follow-up period only. An additional PPD reduction and CAL gain were observed in patients who were given a metronidazole-amoxicillin combination and, to a lesser degree, who were given metronidazole and azithromycin. These effects were more pronounced in patients treated with a metronidazole-amoxicillin combination, especially in those with deep or moderately deep pockets [17]. 

Similar results were found in a systematic review conducted by Haffajee et al. who found similar results, consisting of 26 RCTs and three quasi-experimental studies. A meta-analysis of 22 of these RCTs concluded that the use of systemic antibiotics improved the attachment levels AL (p-value < 0.001) when used as an adjunct to SRP [18]. However, their benefit was termed as “borderline” when used as an adjunct to SRP and surgery or when used as a monotherapy. The CAL gain was observed in chronic and aggressive periodontitis patients, with greater improvements seen in those affected by aggressive periodontitis [18].

CP affects 5%-20% of the population [11]. A systematic review and meta-analysis conducted by Sgolastra et al. included the results of four RCT studies, which evaluated the efficacy of amoxicillin and metronidazole as an adjunctive therapy to SRP in the treatment of CP [11]. The review concluded a significant CAL gain (WMD: 0.21; CI 95%: 0.02-0.4; p < 0.05) with PPD reduction (0.43); CI 95%: 0.24-0.63; p < 0.05). The effect was minimal on the improvement of bleeding on probing (BOP) or suppuration. Thus, the review and meta-analysis supported the adjunctive use of antibiotics (metronidazole and amoxicillin) and SRP compared to SRP alone in treating CP [11]. However, due to limited data available, the role of systemic antibiotics in CP is unclear. Still, they may be considered in specific cases, such as recurrent or refractory cases where the disease does not respond to conventional mechanical debridement [20].

In aggressive periodontitis, systemic antibiotics are more widely accepted as adjunctive therapy. A review by Mendes et al. found significant reductions in probing depths when amoxicillin and metronidazole were combined with SRP, though no significant gain in CAL was observed [12,13]. Current guidelines suggest that the adjunctive use of systemic antibiotics may be considered for specific patient categories, such as younger adults with periodontitis grade C, where a high rate of progression is documented [20]. Rabelo et al. did a systemic review and meta-analysis including all studies until 2014; the results favored systemic antibiotics’ use to cure aggressive periodontitis [21].

Local Antimicrobials in Periodontal Therapy

The latest guidelines advise against using local antimicrobials routinely in periodontal treatment due to limited benefits and resistance concerns [13]. However, they may be considered selectively for persistent deep or recurrent pockets that are unresponsive to SRP and aggressive periodontitis with sites resistant to mechanical treatment. When used as adjuncts to SRP, local antimicrobials can improve clinical outcomes, especially in deep or recurrent cases [13].

A systematic review by Matesanz-Perez et al. evaluated the benefits of local antimicrobials as adjuncts to SRP compared to subgingival mechanical debridement alone. The analysis included data from 56 publications reporting on 52 RCTs, focusing primarily on changes in PPD, with CAL and BOP as secondary outcomes [13]. The studies exhibited significant heterogeneity regarding the types of antimicrobials used and follow-up durations [13]. 

The meta-analysis reported statistically significant reductions in PPD (WMD = 0.407 mm) and gains in CAL (WMD = 0.310 mm) [13]. No significant changes were observed in BOP or plaque index. Tetracycline fibers showed the most substantial PPD reduction (0.727 mm) based on data from four RCTs involving 350 patients. Sustained-release minocycline and doxycycline also demonstrated PPD reductions ranging from 0.5 mm to 0.7 mm.

Chlorhexidine with xanthan gel resulted in the highest CAL gain (0.9 mm), though this finding was based on a single study [13]. Chlorhexidine and locally administered metronidazole showed minimal benefits compared to control groups. Overall, sustained-release local antimicrobials improved clinical outcomes in treating deep pockets and recurrent cases of CP [13]. These results align with previous reviews by Hanes and Purvis et al. and Bonito et al. [13].

A more recent systematic review by Herrera et al., published in March 2020, included 50 studies and evaluated PPD reduction as the primary outcome at six- and nine-month follow-ups. The meta-analysis favored adjunctive local antimicrobials with SRP in treating periodontitis, reporting a PPD reduction (WMD = 0.365 mm) and CAL gain (WMD = 0.263 mm) [14]. No significant adverse side effects were reported with adjunctive local antimicrobial therapy [14].

For a localized site with deep periodontal probing depths, using locally delivered antibiotics (e.g., minocycline microspheres) [22,23] or antimicrobials (e.g., chlorhexidine chips) may be an appropriate consideration [13]. Host modulation therapy has been shown to provide benefits in various studies. When administered at sub-antimicrobial doses, doxycycline can reduce the activity of matrix metalloproteinases (MMPs) in gingival tissues without having any antimicrobial effects [24]. Studies have indicated that using sub-antimicrobial doxycycline as an adjunct to SRP can significantly improve outcomes for patients with periodontitis [24]. Moreover, combining omega-3 fatty acids and 81 mg of acetylsalicylic acid (aspirin) alongside SRP has been associated with notable reductions in probing depths and significant gains in clinical attachment compared to SRP alone [25]. This additive improvement may be attributed to the synergistic effect of promoting the production of protective host mediators like resolvins and the anti-inflammatory actions that help resolve the underlying inflammation [23]. Other therapies that have been explored as adjuncts include antimicrobial photodynamic therapy, laser treatment, probiotics, propolis, and chlorhexidine [23]. Few studies reported adverse effects associated with local antimicrobial delivery. Reported side effects included pain and gingival erythema on the first day, taste disturbances, root sensitivity, fiber or chip dislodgment, headache, fever, and stomatitis [13,14].

Implications for Clinical Practice, Limitations, and Directions for Future Research

Current guidelines emphasize the critical need for antimicrobial stewardship, advising that antibiotics should only be prescribed for severe or refractory cases of infection, where other treatment methods have proven ineffective [11,17,21]. This review further highlights the importance of systemic antimicrobials in managing aggressive or advanced cases of infection, particularly when conventional treatments fail to yield the desired results [16-18]. For patients with chronic or aggressive periodontitis, where deep or recurrent pockets persist despite standard therapy, the use of local antimicrobial agents is recommended. These localized treatments can target the infection more precisely, offering a more effective alternative when systemic antibiotics are not necessary [13]. Furthermore, microbial analysis is strongly advised to guide the selection of the most appropriate antibiotics, ensuring that the chosen treatment is both effective and specific to the infection. Caution is paramount, as inappropriate or broad-spectrum antibiotic use can contribute to the development of antibiotic resistance, posing a significant public health risk [20].

This literature review has several limitations that should be acknowledged. The included studies exhibit high heterogeneity in study designs, antimicrobial types, and follow-up durations, making direct comparisons challenging [23]. Additionally, some studies lack robust long-term data, limiting insights into the sustained effects of antimicrobial therapy in periodontal disease management. Potential biases may also arise from study selection and publication bias, as studies with significant results are more likely to be published. Future research should focus on well-designed, long-term RCTs to establish clearer guidelines on optimal systemic and local antimicrobials in different types and stages of periodontitis [23].

## Conclusions

Currently, limited and low-quality evidence supports the routine use of systemic antimicrobials in nonsurgical periodontal treatment. Antibiotics should be reserved for specific clinical situations, such as aggressive periodontitis or in cases where patients do not respond to standard mechanical therapy. The use of local antimicrobials presents a promising alternative, providing a targeted approach for managing localized disease. This approach offers the potential for fewer systemic side effects and a lower risk of contributing to antimicrobial resistance, making it a more focused treatment option. Local antimicrobials should be considered primarily for deeper pockets or persistent infection sites, and they should be used in conjunction with mechanical debridement to enhance treatment outcomes. Given its proven efficacy, the amoxicillin-metronidazole combination remains the preferred regimen for treating aggressive periodontitis. However, due to concerns about the growing issue of antibiotic resistance, its use should be carefully restricted to severe or refractory cases only. Further high-quality RCTs are needed to better understand the long-term benefits and risks associated with antimicrobial therapy in periodontitis. These studies should focus on clinically relevant outcomes such as CAL, PPD, and BOP to provide more robust data on the long-term effects of antimicrobials in managing periodontitis.
